# Influences of split application and nitrification inhibitor on nitrogen losses, grain yield, and net income for summer maize production

**DOI:** 10.3389/fpls.2022.982373

**Published:** 2022-08-29

**Authors:** Baizhao Ren, Zhentao Ma, Bin Zhao, Peng Liu, Jiwang Zhang

**Affiliations:** State Key Laboratory of Crop Biology, College of Agronomy, Shandong Agricultural University, Tai’an, China

**Keywords:** fertilization method, nitrapyrin, ammonia volatilization, N_2_O emission, nitrogen leaching, grain yield

## Abstract

The application of nitrogen (N) fertilizer combined with nitrification inhibitor is considered to be one of the effective strategies to improve N efficiency and reduce N loss. While the chemical and physical properties of nitrapyrin (CP) in fertilizers have been evaluated to increase N efficiency, a lack of comprehensive evaluation of the effects of adding CP on summer maize yield, environmental benefits and economic income under different fertilization methods. In this study, two fertilization methods were used: split-N application and one-time basal N fertilizer before sowing. The comprehensive effects of N fertilizer with CP on N loss (NH_3_ volatilization, NO_3_^–^ leaching, and N_2_O emissions), N efficiency, yield and profit under two N application methods were explored. Results showed that under the two N application methods, N fertilizer with CP treatment increased the N efficiency and yield (+3.4%∼+5.7%), significantly reduced the soil NO_3_^–^-N content and N_2_O emissions, while increased NH_3_ volatilization. Especially, the increase amplitude of NH_3_ was much less than the decrease amplitude of N_2_O induced by adding CP. Although split-N application could achieve higher yield and N efficiency, N_2_O emissions and NH_3_ volatilization also increased. However, the T1 + N (one-time basal N fertilizer before sowing mixed with CP) achieved the same yield level as T2 treatment (split-N application). Taking agronomic, economic and environmental benefits into consideration, one-time basal N fertilizer before sowing mixed with CP could ensure the target yield, increase economic benefits, maintain soil N content, and reduce N losses. Therefore, optimizing N management is essential to the sustainable development of agriculture.

## Introduction

Global food demand requires increased uses of fertilizers, although the use of chemical fertilizer guarantees the high crop yield, it causes low utilization rate, the loss of fertilizer, and a series of environmental pollution problems (Hao et al., 2019). Nitrogen (N) is one of the most restricted nutrients in the formation of maize yield, but excessive and uncoordinated fertilization methods often result in low nitrogen use efficiency (NUE) (Nasielski et al., 2020). Therefore, management practices need to be improved to increase crop productivity and reduce environmental hazards.

In the growing season, split-N application is usually used to meet the N demand of maize to the greatest extent to obtain higher yields (Rahman et al., 2011; Wang et al., 2016). Compared with one-time application of N fertilizer, split-N application could significantly prolong the active period of N accumulation in summer maize and significantly increase the NUE (Lv et al., 2011; Lu et al., 2021). However, the shortage of agricultural workers and higher-cost inputs often limit implementation. Therefore, measures such as one-time base application of urea are usually used in maize production (Lu et al., 2021). After urea is applied to the soil, it is hydrolyzed to ammonium (NH_4_^+^) and then quickly converted to nitrate (NO_3_^–^). Heavy rainfall often causes a large amount of NO_3_^–^ leaching into the deep soil layer, which leads to insufficient N to meet the maize demand in the late season (Azad et al., 2019; Fernandez et al., 2020). In order to reduce N loss as much as possible, some technologies, such as the application of controlled-release N fertilizer, improve NUE by controlling the release time of N (Fan et al., 2021). But the positive effects may be limited due to a slowed release of N from controlled-release N fertilizers, especially during early season (Qu et al., 2020). Adding nitrification inhibitors is considered to be one of the effective ways to increase crop yield and NUE. Nitrification inhibitors inhibit soil nitrification, and directly affecting nitrifying bacteria community and nitrification activity, keeping N in the form of NH_4_^+^ in soil for a long period, and thereby reducing leaching loss of nitrate N and denitrification of soil (Menendez et al., 2006; Sun et al., 2008; Monge-Muñoz et al., 2021).

Nitrapyrin, short for 2-chloro-6-(trichloromethyl) pyridine (CP, N-serve), is a white crystalline solid substance, almost insoluble in water, soluble in organic solvents (Goring, 1962; Borzouei et al., 2021). It is one of the most widely studied and effective nitrification inhibitors. In some regional trials, adding CP significantly increased yield and NUE (Sun et al., 2015). However, a meta-analysis showed that the addition of CP changed the crop yield by –20∼207% (Wolt, 2004). It can be seen that the effectiveness of CP is variable. CP inhibits the activity of soil ammonia oxidizing bacteria and retard the conversion of NH_4_^+^ into NO_2_^–^ (the first step of nitrification), thereby keeping the fertilizer in the form of NH_4_^+^. However, higher NH_4_^+^ in the soil may increase ammonia volatilization. In a multiyear rice study, the urea with CP increased the NH_3_ volatilization by 7% compared to the urea treatment without CP (Sun et al., 2015). Conversely, CP targets nitrification and limits the formation of NO_3_^–^ (the substrate for denitrification), so it has potential to reduce N_2_O emissions. It is reported that CP reduces greenhouse gas emissions by 21∼70% compared with treatment without CP (Akiyama et al., 2010). The evaluation results of the effects of using nitrification inhibitors on N_2_O emissions and NH_3_ volatilization are not consistent.

Most studies have addressed the effect of N management on increasing yield, reducing N_2_O emissions and NH_3_ volatilization, but little research has been directed toward how to balance the relationship between yield and N loss, and to resolve the contradiction between economic income and ecological effects. Therefore, it is necessary to reconcile production goals with environmental costs, and choose a reasonable N management strategy. In this study, a comprehensive evaluation of the effects of adding CP on summer maize yield, environmental benefits and economic income under different fertilization methods was conducted. It may provide scientific basis for high-yield and high-efficiency cultivation of summer maize and reduce environmental pollution. We hypothesize that the use of disposable basal application of urea with adding CP can achieve the same or higher yield and NUE as the split-N application, and reduce environmental pollution. Specially, the aims of this study were to: (i) investigate the effects of adding CP on yield and N efficiency of summer maize, (ii) illustrate the relationships among N fertilizer adding CP, N_2_O emissions, NH_3_ volatilization, and NO_3_^–^-N/NH_4_^+^-N accumulation of farmland soil, (iii) explore better N application methods for the environment and economic income.

## Materials and methods

### Plant materials and experimental design

This study was carried out at the experimental farm (36°10’N, 117°04’E, 151 m a.s.l.) maintained by the State Key Laboratory of Crop Biology of Shandong Agricultural University, China in 2016 and 2017. The region was characterized by brown loam soils and a temperate continental monsoon climate with mean annual temperature of approximately 13°C. The rainfall mainly focused on June, July and August, with an average rainfall of 697 mm in the two maize seasons. Soil physical and chemical properties (0–20 cm) are as listed: (i) pH is 7.2; (ii) organic matter is 10.7 g kg^–1^; (iii) total N is 0.9 g kg^–1^; (iv) available phosphorus is 50.7 mg kg^–1^; and (v) available potassium is 86.2 mg kg^–1^. Denghai618 (DH618), a commonly grown maize hybrid, was used for this experiment. Maize seeds were sown on 16-June 2016 and 20-June 2017 at a density of 60,000 plants ha^–1^ and 75,000 plants ha^–1^, respectively.

The nitrification inhibitor was 2-chloro-6-(trichloromethyl) pyridine (CP), supplied by Dow AgroSciences (Indianapolis, IN, United States). Experimental treatment (length 10 m* width 6 m) was as follow: one-time basal N fertilizer before sowing (T1, 210 kg ha^–1^ N), one-time basal N fertilizer before sowing mixed with CP (T1 + N), split-N application (T2, 105 kg ha^–1^ N was applied before sowing, and the other N was applied at the 10th leaf stage), and split-N application mixed with CP (T2 + N). Urea was used as the N source, and the N application rate was 210 kg ha^–1^ N in all treatments. Both basal and topdressing N fertilizer supplements were applied in bands near the plant row incorporated into the soil *via* plowing. The rate of CP was 0.24% of the rate of urea-N application, the mixture was obtained by physically mixing it with urea. Each treatment was replicated three times. 84 kg ha^–1^ P_2_O_5_ (calcium superphosphate, 17%) and 168 kg ha^–1^ K_2_O (Potassium chloride, 60%) were applied to all plots before sowing. Supplemental irrigation was not applied during the summer maize seasons except for one use after sowing. Micro-sprinkler irrigation was used with a water consumption of about 30 mm. During the growing period of maize, good field management was practiced according to the production habits of local farmers.

### Soil N_2_O fluxes measurements

The nitrous oxide (N_2_O) was collected and analyzed by closed-chamber gas chromatography. Three chambers with a volume of 35 cm length × 35 cm width × 20 cm height were set per for each treatment and placed before each sampling. The closed chamber was enclosed with plastic sheets (sponge material and aluminum foil on the outside) and an air vent was installed in the middle of chamber. A pedestal was placed under the chamber and sealed with water. The gas samples (50 mL) were collected once every one days within a week after fertilization, and then every 7–10 days (1–2 times a week, then every 7–10 days after fertilization in 2017) for collection at 8:00–11:00 a.m. using glass syringes from chamber headspace 0, 10, 20, and 30 min after soil sample was covered. The gas samples were analyzed for N_2_O concentration within 36 h after sampling by an Agilent GC7890 gas chromatograph (Agilent, Santa Clara, CA, United States) equipped with an electron capture detector (ECD). The N_2_O emissions flux was calculated as follow (Yu et al., 2022):


(1) $$J=\frac{d\,c}{d\,t}\times \frac{M\,P}{V\,0}\times \frac{T\,0\,H}{P\,0\,T}$$

where *J* is N_2_O emissions flux (mg m^–2^ h^–1^), *dc/dt* is the change in gas concentration per unit time (mg m^–3^ h^–1^). *M* is the molar mass of the gas (mg mol^–1^), *P* is atmospheric pressure (KPa), *T* is the absolute temperature (*K*), *H* is the height of the chamber (m), *V0* is the gas molar volume under standard conditions (m^3^ mol^–1^), *T0* is the absolute air temperature under standard conditions (*K*), *P0* is the atmospheric pressure under standard conditions (KPa).

### Ammonia volatilization

Five vented-chamber devices for NH_3_ volatilization measurements were placed for each plot (Zhao et al., 2013). The vented chamber consisted of a gray round polyvinyl chloride (PVC) tubing (15 cm internal diameter and 10 cm high) and two round sponges (16 cm in diameter and 2 cm in thickness). The sponges were presoaked with a 15 mL phosphate-glycerol solution (50 mL analytical phosphate and 40 mL glycerol diluted to 1,000 mL with pure water) and then inserted into each chamber.

In summer maize-growing season, the sponges were replaced between 8:00 a.m. and 11:00 a.m. daily after fertilization during the first week and every 2–3 days during the second and third week. Ammonia in the phosphate solution in each sponge inside the vented chamber was extracted with 300 mL of 1 M KCl after 1 h of oscillation. Ammonium quantities were measured using the micro-Kjeldahl method (CN61M/KDY-9820, Beijing, China). NH_3_ volatilization from the soil was estimated by the following formula (Li et al., 2007):


(2) $$\text{NV}=\frac{M}{A\,D}\times 10^{-2}$$

where NV is the NH_3_ volatilization (kg N ha^–1^ d^–1^), *M* is the NH_3_ captured by the vented chamber during each sampling (mg N), *A* is the cross-section area of the round chamber (m^2^), *D* is the duration of each sampling (days).

### Soil NH_4_^+^-N and NO_3_^–^-N

In each plot, three replicate soil samples were collected every 7 days from the start of basal N fertilizer supplement to the six-leaf stage (V6), topdressing N fertilizer supplement to the tasseling stage (VT), and then every 14 days until the maturity stage (R6) in 2016. And soil samples were taken at V6, VT, and R6 stage in 2017. The samples were divided into three layers (60 cm length by 20 cm depth). Soil NH_4_^+^-N and NO_3_^–^-N were extracted with 1 M KCl and measured by AA3 Continuous Flow Analytical System.

### Nitrogen efficiency

Five plant samples were obtained from each plot at R6 stage. All samples were separated into stem, leaf, and ear, dried at 60°C in a force-draft oven (DHG-9420A, Bilon Instruments Co. Ltd., Shanghai, China) to a constant weight. Total N was measured using the Kjeldahl method. Partial factor productivity from applied nitrogen (PFPN, kg kg^–1^), agronomic efficiency of applied nitrogen (AEN, kg kg^–1^), nitrogen recovery efficiency (REN, kg kg^–1^) and soil nitrogen dependency rate (SNDR, %) were calculated as follow (Wu et al., 2008):


(3) $$\text{PFPN}=\frac{\text{YF}}{\text{NA}}$$

(4) $$\text{AEN}=\frac{\text{YF}-\text{YC}}{\text{NA}}$$

(5) $$\text{SNDR}=\frac{\text{TC}}{\text{TN}}\times 100$$

(6) $$\text{REN}=\frac{\text{TN}-\text{TC}}{\text{NA}}$$

where YF is the grain yield in the fertilized plot (kg), YC is the grain yield in the control plot (kg), NA is the amount of applied N (kg), TC is the total N uptake by plant in the control plot (kg), TN is the total N uptake by plant in the fertilized plot (kg).

### Crop yield

Grain yield and ear traits were analyzed by harvesting the three central rows of each plot in 2016–2017. AT R6 stage, 30 consecutive plants per row were harvested to measure summer maize yield (moisture content is approximately 14%, GB/T 29890-2013).

### Net income

Environmental cost (EC, $ kg^–1^) was the cost of N emissions pollution caused by N fertilization, following the calculation of Yin et al. (2019):


(7) EC=N⁢2⁢O×⁢11.2⁢+NH3×⁢2.11

where EC refer to the environmental damage costs caused by soil acidification and climate warming caused by N_2_O and eutrophication caused by NH_3_ volatilization. The pollution cost values of N_2_O-N and NH_3_-N are $11.2 and $2.11 kg^–1^, respectively.

Net income (NI, $ ha^–1^) was calculated as follows (Zhang et al., 2018):


(8) NI=GI-AC-EC

where GI is the grain income (determined by grain yield multiplying the grain price), AC is the agronomic cost.

### Statistical analysis

The statistical analyses were performed using SPSS 17.0 (SPSS Inc., Chicago, IL, United States). All data were subjected to one-way analyses of variance (ANOVA) followed by mean comparisons using the least significant difference (LSD, *P* ≤ 0.05). All the figures were produced using Sigma Plot 14.0 (Systat Software Inc., CA, United States).

## Results

### Grain yield

Compared with one-time base application of N fertilizer, the split-N application treatment significantly increased the grain yield. The grain yield of T2 was 2.5% higher than that of T1, and the T2 + N treatment was 4.7% higher than T1 + N. Furthermore, the fertilized treatments with the CP exhibited higher grain yield than treatments without the CP under the two fertilization methods. The yield increase of CP treatment was mainly to the improvement of grain number per spike and 1000-grain weight of summer maize by 2.2% and 1.4% on average, compared to those of no CP treatment, while the differences were not significant. In particular, compared with the split-N application treatment (T2), the one-time base application of N fertilizer with CP (T1 + N) increased the grain yield (the grain yield of T1 + N treatment was higher than that of T2 for 1 year, while there was no significant difference in 2016) (Table 1).

**TABLE 1:** Effect of nitrogen with CP on grain yield and yield components of summer maize under different fertilization methods.

Year	Treatment	Ears (No./ha)	Grains per ear (No./ear)	1,000-kernel weight (g)	Grain yield (kg/ha)
2016	CK	52479b^↕^	540c	356d	10089d
	T1	57098a	583b	372b	12383c
	T1 + N	57527a	588b	375ab	12685b
	T2	57360a	585b	378a	12684b
	T2 + N	57622a	608a	374ab	13103a
2017	CK	72384a	394d	373d	10642e
	T1	71006a	449c	396c	12633d
	T1 + N	71078a	455b	407b	13177b
	T2	70673a	456b	402b	12955c
	T2 + N	72305a	468a	414a	13994a

CK, no nitrogen treatment; T1, one-time basal N fertilizer before sowing; T1 + N, one-time basal N fertilizer before sowing mixed with CP; T2, split-N application; T2 + N, split-N application mixed with CP.

^↕^Values fallowed by a different small letter within a column are significantly different (P < 0.05).

### N_2_O emission

**FIGURE 1:**
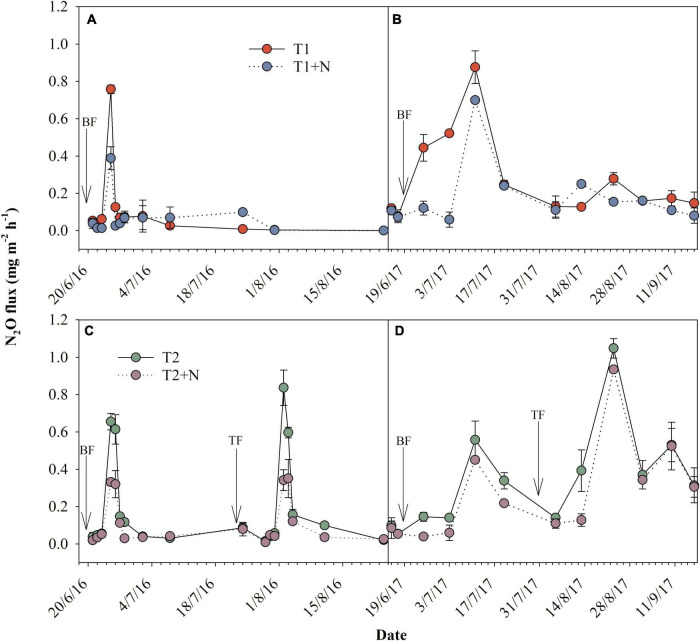
Dynamics of N_2_O volatilization emission rates. **(A,B)** T1, one-time basal N fertilizer before sowing; T1 + N, one-time basal N fertilizer before sowing mixed with CP; **(C,D)** T2, split-N application; T2 + N, split-N application mixed with CP; BF, basal N fertilizer supplement; TF, topdressing N fertilizer supplement. The downward arrow indicates fertilization.

As shown in Figure 1, N_2_O emission rates peaked within 10–20 days after applying the base fertilizer and topdressing, and then decreased. The first peak value of the split-N application treatment was significantly lower than the one-time base application of N fertilizer, but the second increased significantly. In addition, the cumulative N_2_O emission fluxes of split-N application treatment in two maize seasons were significantly higher than the one-time base application of N fertilizer. Obviously, under the two N application methods, the N_2_O emission peak value of the CP treatment was significantly reduced. Compared with T1, the peak value of T1 + N treatment decreased by 48.6 and 20.2% in 2016 and 2017, respectively; while the first peak value of T2 + N treatment was reduced by 49.5 and 19.4%, respectively, compared with T2, and the second was reduced by 59.2 and 10.8%. Similarly, the cumulative N_2_O emission fluxes of the fertilized treatments with the CP were also significantly decreased. In addition, the cumulative N_2_O emission fluxes of the one-time base application of N fertilizer with the CP was significantly lower than other treatments.

### NH_3_ emission

**FIGURE 2:**
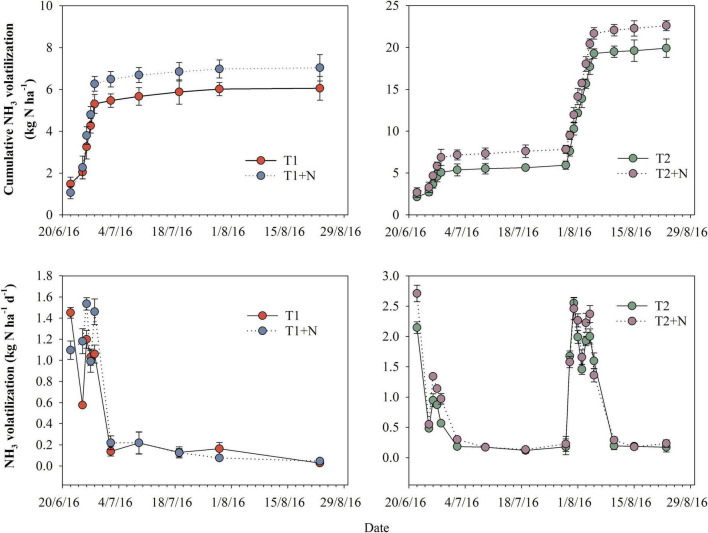
Dynamics of NH_3_ volatilization emission rates and cumulative NH_3_ volatilization. T1, one-time basal N fertilizer before sowing; T1 + N, one-time basal N fertilizer before sowing mixed with CP; T2, split-N application; T2 + N, split-N application mixed with CP.

Split-N application significantly increased NH_3_ volatilization rate, especially after top dressing. Under the two N application methods, on CP addition, the peak value and duration of NH_3_ volatilization increased, and the accumulation of NH_3_ volatilization was also higher than the single N application treatment. The accumulation of NH_3_ volatilization for T1 + N was 15.6% greater than T1 treatment, and the T2 + N treatment was increased by 12.9%, compared to T2. However, the cumulative NH_3_ volatilization of the one-time base application of N fertilizer with CP treatment (T1 + N) was lower than that of the split-N application treatment (T2) (Figure 2).

### Soil NH_4_^+^-N and NO_3_^–^-N contents

**FIGURE 3:**
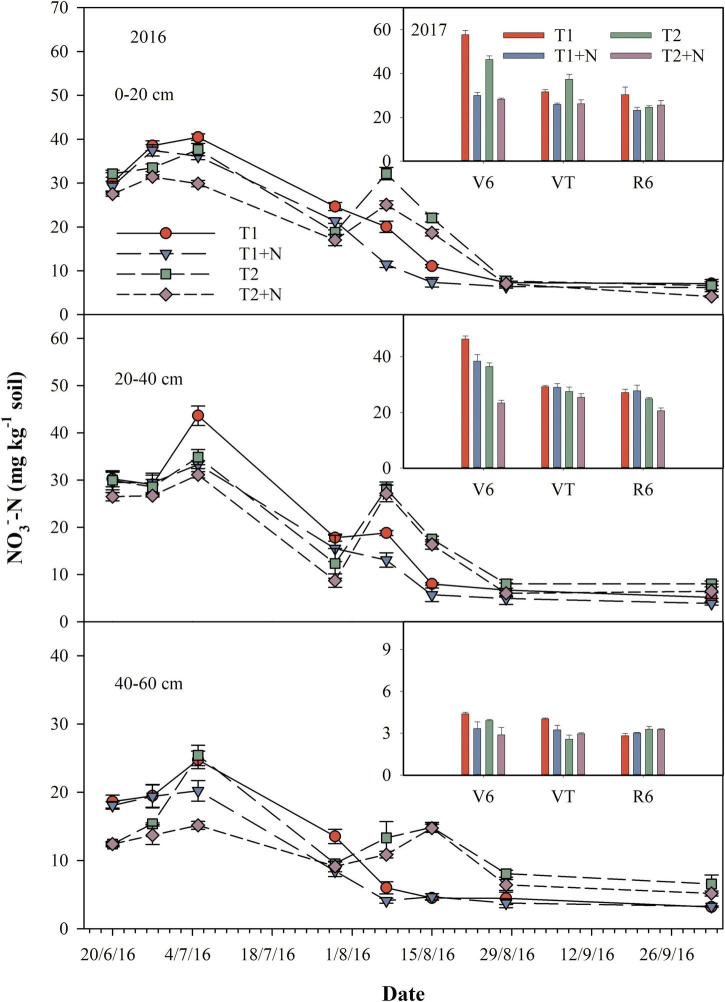
Dynamics of soil NO_3_^–^-N content. T1, one-time basal N fertilizer before sowing; T1 + N, one-time basal N fertilizer before sowing mixed with CP; T2, split-N application; T2 + N, split-N application mixed with CP; V6, six-leaf stage (12/7/2017); VT, tasseling stage (5/8/2017); R6, maturity stage (3/10/2017).

The soil NO_3_^–^-N content of the one-time base application of N fertilizer treatment increased first and then decreased, while the split-N application treatment had two obvious peak value. Under the two N application methods, on CP addition, the NO_3_^–^-N content in the different soil layers was lower than the single N application treatment. The content of soil NO_3_^–^-N for T1 + N was decreased by 13.1, 15.1, and 13.4% in 0–20, 20–40, and 40–60 cm, compared to those of T1, respectively. And the T2 + N treatment was decreased by 15.6, 11.0, and 17.0%, compared to those of T2, respectively. The results of different stages of maize in 2017 showed that the adding CP treatment significantly reduced NO_3_^–^-N content in the 0–20 cm soil layer at V6 and VT, but there was no significant difference in the 40–60 cm soil layer at R6 stage, compared to the single N application treatment (Figure 3).

**FIGURE 4:**
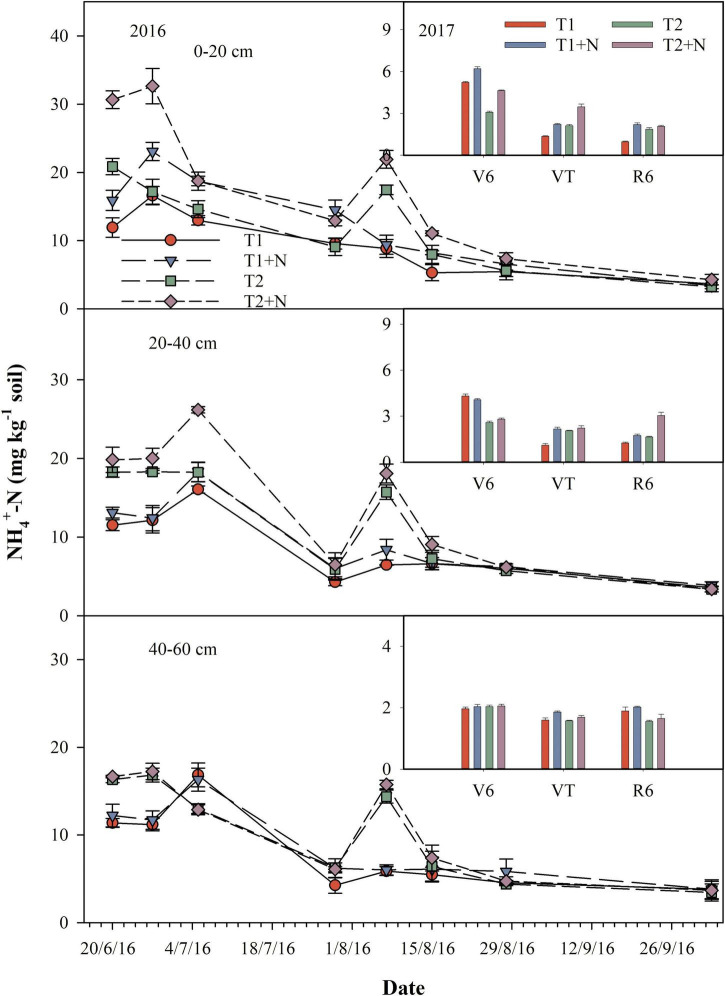
Dynamics of soil NH_4_^+^-N content. T1, one-time basal N fertilizer before sowing; T1 + N, one-time basal N fertilizer before sowing mixed with CP; T2, split-N application; T2 + N, split-N application mixed with CP; V6, six-leaf stage (12/7/2017); VT, tasseling stage (5/8/2017); R6, maturity stage (3/10/2017).

After fertilization, the content of soil NH_4_^+^-N increased firstly and then decreased. The spatial and temporal distribution of NH_4_^+^-N in soil was changed by split-N application, and the retention time of NH_4_^+^-N in soil was effectively improved, compared to that of one-time base application of N fertilizer. In addition, the content of NH_4_^+^-N in the 0–40 cm soil layer increased significantly when the CP was added, but there was no significant difference in the 40–60 cm soil layer. The content of soil NH_4_^+^-N for T1 + N was increased by 34.2, 12.4, and 7.6% in 0–20, 20–40, and 40–60 cm, compared to those of T1, respectively. However, the content of soil NH_4_^+^-N for T2 + N was increased by 45.4, 17.7, and 4.1% in 0–20, 20–40, and 40–60 cm, compared to those of T2, respectively (Figure 4).

### Nitrogen efficiency

Split-N application significantly increased the nitrogen accumulation (NA), nitrogen partial factor productivity (PFPN), nitrogen agronomic efficiency (AEN), and nitrogen recovery efficiency (REN) of summer maize. For all the N application treatments, the addition of CP obtained higher N efficiency. The PFPN, AEN, and REN of T1 + N were increased by 3.4, 20.2 and 162.7%, respectively, compared with T1. Compared with T2, the T2 + N treatment were increased by 5.7, 30.5, and 46.0%, respectively. However, the soil nitrogen dependency rate (SNDR) of CP treatment was decreased significantly, with an average decrease of 13.1%, compared with that of no CP treatment. Furthermore, compared with T2, the NA and REN of TI + N treatment increased by 8.8 and 36.8% respectively in 2016, while there was no significant difference in 2017 (Table 2).

**TABLE 2:** Effect of nitrogen with CP on nitrogen efficiency of summer maize under different fertilization methods.

Year	Treatment	NA (g/plant)	PFPN (kg/kg)	AEN (kg/kg)	SNDR (%)	REN (%)
2016	CK	2.61d^↕^	–	–	–	–
	T1	2.98c	58.97c	10.93c	87.74a	10.43c
	T1 + N	3.71a	60.40b	12.36b	70.43c	31.33a
	T2	3.41b	60.40b	12.36b	76.51b	22.90b
	T2 + N	3.78a	62.39a	14.35a	69.15c	33.29a
2017	CK	2.60d	–	–	–	–
	T1	2.96c	60.16c	9.48c	87.84a	12.86c
	T1 + N	3.41b	62.75b	12.07b	76.25b	28.93b
	T2	3.37b	61.69b	11.01b	77.15b	27.50b
	T2 + N	3.73a	66.64a	15.96a	69.71c	40.36a

CK, no nitrogen treatment; T1, one-time basal N fertilizer before sowing; T1 + N, one-time basal N fertilizer before sowing mixed with CP; T2, split-N application; T2 + N, split-N application mixed with CP; NA, nitrogen accumulatio; PFPN, nitrogen partial factor productivity; AEN, nitrogen agronomic efficiency; SNDR, soil nitrogen dependency rate; REN, nitrogen recovery efficiency.

^↕^Values fallowed by a different small letter within a column are significantly different (P < 0.05).

### Economic and environmental benefits

Compared with the one-time base application of N fertilizer treatment, split-N application significantly increased the yield value, while also increasing labor input and environmental costs. Under the two fertilization methods, the net income of the treatment with CP was significantly higher than the single N application treatment. Compared with T1, the net income of T1 + N increased by 4.1%, and the T2 + N treatment enhanced by 7.1% compared with T2. Overall, the net income of T2 + N was significantly higher than that of other treatments, while there was no significant difference between T1 and T2 treatments (Table 3).

**TABLE 3:** Economic benefits under different nitrogen application treatments.

Treatment	Yield value ($ ha^–1^)	Economic input ($ ha^–1^)			Environmental costs ($ ha^–1^)		Net income ($ ha^–1^)
			
		CP	Labor	Others	N_2_O	NH_3_	
T1	5498.52c^↕^	–	453.34	440.86	54.32b	121.09d	4428.91c
T1 + N	5684.47b	2.98	453.34	440.86	35.39c	139.99c	4611.90b
T2	5635.45b	–	491.61	440.86	75.66a	217.61b	4409.70c
T2 + N	5955.92a	2.98	491.61	440.86	53.02b	245.60a	4721.85a

^↕^Different letters within a column indicate statistically significant differences (P < 0.05).

## Discussion

Reasonable N application is considered to be one of the most effective management strategies to increase crop yield and N efficiency. It is necessary to consider the appropriate N source, application rate and time during crop growth to increase yield and N efficiency, and reduce production costs and environmental pollution (Abbasi et al., 2012; Schulte-Uebbing and Vries, 2021). Therefore, it is usually recommended to apply fertilizer in batches as a way to increase output and reduce N loss in production (Xu et al., 2021). Compared with single N application before sowing, split N application could increase grain yield and NUE (Sangoi et al., 2007). In this study, compared with the one-time basal application before planting, the grain yield of summer maize increased by divided fertilization, especially the 1,000-grain weight increased (Table 1), which was similar to the previous study (Amanullah and Shah, 2010; Guo et al., 2016). The increase in yield caused by the split-N application may be due to the acquisition of more effective N in the late growth period of summer maize. However, the output was enhanced under the two fertilization measures after adding CP. Obviously, there was no significant difference between the one-time base application of urea with CP added before sowing and the split-N application treatment. This may be related to the increase in NUE after adding CP. Sun et al. (2015) reported that adding CP to rice could save 60 kg N ha^–1^. Similarly, the results of N efficiency also showed that N accumulation, AEN, REN, and PFPN were significantly improved after adding CP (Table 2).

In crop production, the loss of N is difficult to effectively avoid. Although it has been found in most studies that adding a nitrification inhibitor would minimize N loss. However, there were also opposite research results, especially the results of N_2_O emissions and ammonia volatilization were not the same. Nitrapyrin mainly acted on nitrite oxidizing bacteria (Nitrobacter) to participate in the oxidation of NO_2_^–^ to NO_3_^–^ process, which was the second step in nitrification, but as long as one step of the reaction was inhibited, the whole nitrification was inhibited (Wu et al., 2008). Previous studies showed that nitrification inhibitors could slow down soil nitrification rate by acting on soil microorganisms, reduce the accumulation of NO_2_^–^-N, and ultimately achieved the goal of reducing N_2_O emissions (Ju et al., 2011; Hu et al., 2013).

Nitrification inhibitors reduced soil N_2_O emissions, while increased soil NH_3_ volatilization at the same time (Freney et al., 1995; Parkin and Hatfield, 2010; Rex and Tony, 2013). Our studies showed that NH_4_^+^-N concentrations after applying CP were higher in 0–20 and 20–40 cm soil layers compared to concentrations in no CP treatment, whereas NO_3_^–^-N concentrations in all soil layers (0–20, 20–40, and 40–60 cm) were lower in CP treatment (Figures 3, 4). This result might be due to the presence of nitrification inhibitors, which prevented the transformation of NH_4_^+^-N into NO_3_^–^-N, and maintaining N in the form of NH_4_^+^-N in soil for a longer time period. Since N was retained in the form of less mobile NH_4_^+^-N, the rate of leaching was significantly slowed, and thus reduced N leaching and increased NUE (Rahmatullah et al., 2010). Nitrate leaching losses is mainly determined by the concentration of NO_3_^–^-N in the soil solution and the amount of soil leachate (Vibart et al., 2016). Similarly, split fertilization also slowed down the leaching rate of NO_3_^–^-N in shallow soils and prolonged the effectiveness of N. This also explained the increase in grain yield, although it also caused an increase in N_2_O emissions (Figure 1).

After adding CP, soil nitrification was inhibited, the content of NH_4_^+^-N in the soil was at a high level, resulting in the loss of NH_4_^+^-N in the form of NH_3_ volatilization (+12.9%∼+15.6%) (Figure 2). However, the increase amplitude of NH_3_ was much less than that of N_2_O (–29.9∼-34.8%) induced by applying CP. But it did not reduce environmental costs (Table 3). The reason for this may be that we did not consider other costs such as NO_3_^–^-N leaching in the soil. In addition, the net income obtained by adding CP treatment increased significantly. Therefore, adding CP in actual production may have lower environmental costs and higher benefits. Especially, our results also showed that compared with the split-N application treatment, the net benefit of the one-time base application of N fertilizer with CP increased significantly. It can be seen that optimizing N fertilizer management would significantly improve crop production.

## Conclusion

Optimizing N fertilizer management is essential to increase agricultural productivity and economic benefits, improve environmental pollution and human health. Our research aggregated N application methods, N losses, output value and benefits, which may provide an effective method for balancing the relationship between agronomic, economic, and environmental benefits. In this study, although split-N application could achieve higher yield, it also caused higher N_2_O emissions and NH_3_ volatilization. Nitrapyrin was conducive to alleviate N_2_O emission and N leaching loss, improving N efficiency, and increasing grain yield of summer maize. One-time basic application of N fertilizer with nitrapyrin was an efficient fertilization method, which achieved the same or even higher yield level as that of split-N application, reduced environmental pollution and obtained better economic value.

## Data availability statement

The original contributions presented in this study are included in the article/supplementary material, further inquiries can be directed to the corresponding author.

## Author contributions

BR: data curation, writing—original draft, visualization, and investigation. ZM: writing—review and editing. PL and BZ: supervision. JZ: conceptualization, writing—review and editing, and funding acquisition. All authors contributed to the article and approved the submitted version.
